# Complement *C3* gene polymorphisms are associated with lipid levels, but not the risk of coronary artery disease: a case-control study

**DOI:** 10.1186/s12944-019-1163-8

**Published:** 2019-12-11

**Authors:** Gaojun Cai, Li Li, Yifei Chen, Haomin Huang, Lei Yu, Lianhong Xu

**Affiliations:** 1 Department of Cardiology, Wujin hospital affiliated with Jiangsu University, the Wujin Clinical college of Xuzhou Medical University, Changzhou, Jiangsu Province, 213017 China; 2 Department of Emergency, the affiliated hospital of Yangzhou university, Yangzhou, Jiangsu Province, 225001 China; 3 Department of laboratory, Wujin hospital affiliated with Jiangsu University, the Wujin Clinical college of Xuzhou Medical University, Changzhou, Jiangsu Province, 213017 China

**Keywords:** Coronary artery disease, Complement C3, Gene, Polymorphism, Haplotype

## Abstract

**Background:**

Coronary artery disease (CAD) is the leading cause of mortality and morbidity worldwide. Previous studies have shown that complement component 3 (C3) is associated with atherosclerosis and cardiovascular risk factors.

**Methods:**

We conducted this study to evaluate the associations between tagSNPs in the *C3* gene locus and the CAD susceptibility and lipid levels in the Chinese population. A hospital-based case-control study, including 1017 subjects (580 CAD patients and 437 non-CAD controls), was conducted. TagSNPs in the *C3* gene were searched and genotyped by using the polymerase chain reaction-ligase detection reaction method.

**Results:**

The C3 levels were positively associated with the low-density lipoprotein cholesterol (LDL-C) levels (r = 0.269, *P* = 0.001). Compared with those in controls, the serum C3 levels in CAD patients were significantly higher (Control: 0.94 + 0.14 g/l; CAD: 1.10 + 0.19 g/l, *P* < 0.001). No significant differences in genotype or allele frequencies were observed between CAD patients and controls. The minor T allele of rs2287848 was associated with low apolipoprotein A1 (ApoA1) levels in controls (Bonferroni corrected P, Pc = 0.032). Linkage disequilibrium and haplotype analysis established two haplotype blocks (Block1: rs344555-rs2277984, Block 2: rs2287848-rs11672613) and six haplotypes. No significant associations between haplotypes and the risk of CAD were observed (all Pc > 0.05).

**Conclusions:**

The results revealed that *C3* gene polymorphisms were associated with the lipid levels, but not CAD susceptibility in the Chinese population.

## Introduction

Coronary artery disease (CAD) is the leading cause of mortality and morbidity in developed countries, as well as in developing countries [1]. China is representative of a developing country. Recently, a report on cardiovascular disease (CVD) revealed that approximately one in five Chinese adults is afflicted by CVD, and that the number of CAD patients is more than 11 million [2]. With the increasing trend of aging in China, the incidence of CAD is bound to increase. Growing evidence suggests that CAD is a multi-factorial condition that is determined by environmental and genetic factors [3].

In recent years, studies have found that activation of complement system plays an important role in the pathogenesis of CAD [4]. The third complement component (C3), mainly secreted by liver and adipose tissue, is the central component of the complement system and plays a crucial role in the immune system. Activation of C3 is the most important step for the biological activity of the complement system. A high fish oil diet promotes the production of the membrane attack complex and increases the levels of various complement proteins in vivo, including C3 [5]. Previous studies have shown that C3 is associated with atherosclerosis and cardiovascular risk factors. Increased deposition of C3 within the intima of atherosclerotic lesions suggests that complement may play a direct functional role in atherosclerosis. Preclinical and clinical evidence suggested that complement C3 might be a biomarker of insulin resistance and cardio-metabolic diseases [6–8]. Bratti et al. revealed that the C3 level was positively correlated with body mass index and significantly decreased with weight loss after bariatric surgery. Jiang et al. found that in CAD patients, serum C3 was significantly higher than in controls, and was positively associated with the severity of CAD [9, 10].

The *C3* gene is located in human chromosome 19p13.3–2 and contains 41 exons. Although the relationship between gene polymorphisms and CAD risk has been investigated extensively, studies focused on *C3* variants were relatively few and the results were inconsistent. A mutation in the *C3* gene could result in an increase in circulating C3 concentrations and was related to dyslipidaemia and cardiovascular disease [11]. In 2015, Nsaiba et al. found that schizophrenic patients with the rs2230199 GG genotype had higher total cholesterol (TC) and, lower density lipoprotein cholesterol (LDL-C) and C3 levels compared with those with the CC genotype [11]. In addition, a strong positive correlation was found between the *C3* polymorphism and myocardial infarction in a Tunisian population [12].

To the best of our knowledge, no study has thoroughly investigated the effect of *C3* gene polymorphisms on CAD and lipid profiles in Chinese population. Therefore, we performed this hospital-based case-control study to explore the relationship between *C3* tagSNPs and the lipid levels and CAD risk in a Chinese population.

## Methods

### Subjects

From October 2012 to July 2017, a total of 1017 unrelated adult subjects were enrolled in this hospital-based case-control study from the Department of Cardiology in Wujin Hospital affiliated with Jiangsu University. Of the 1017 subjects, 580 were CAD patients (410 males and 170 females, mean age of 64.16 ± 9.93 years) and 437 were non-CAD controls (227 males and 210 females, mean age of 61.26 ± 9.35 years). All participants were Chinese and underwent a coronary angiography examination. Coronary angiograms were evaluated by at least two experienced cardiologists. CAD was defined as a stenosis diameter greater than 50% in at least one major coronary vessel (left main, left anterior descending, left circumflex, right coronary artery, and large branches). Non-CAD controls, selected from individuals admitted to the hospital to rule out CAD, also underwent a coronary angiography examination. The luminal stenosis of all of the major coronary arteries must be less than 50%. Diagnoses of EH and DM were described in a previous study **[**13]**.** In addition, individuals with rheumatic disease, asthma, infection, malignancy, serious kidney or hepatic disease were excluded from this study.

Written informed consent was obtained from all included participants. The study protocol conformed to the ethical guidelines of the Declaration of Helsinki and was approved by the Ethics Committee of Wujin Hospital affiliated with Jiangsu University.

### Selection of C3 gene polymorphisms

TagSNPs of the *C3* gene were searched in the International HapMap Project database (http://hapmap.ncbi.nlm.nih.gov/cgi-perl/gbrowse/hapmap3r2_B36/). The detailed strategy was as follows: Population: CHB; Pairwise methods: Tagger Pairwise; RSquare cut off: 0.80; and MAF cut off: 0.10. In total, eight SNPs met the requirements and were included in the present study (rs2250656, rs344555, rs11672613, rs2287848, rs7257062, rs2230205, rs2277984, and rs2241393). The detailed information of the eight SNPs is shown in Additional file 1: Table S1.

### DNA genotyping

After overnight fasting, peripheral venous blood (2 ml) was collected from each patient. The methods for genomic DNA extraction and storage were described in our previous study [14]. The polymerase chain reaction-ligase detection reaction (PCR-LDR) method was performed to identify the genotypes of each polymorphism. The PCR primers and LDR probes were designed with Primer 3 online software (Version 0.4.0) (http://frodo.wi.mit.edu/), and Oligo software (Version 6.31) (Molecular Biology Insights Inc., USA), respectively. Information regarding the PCR primers and LDR probes is provided in Additional file 1: Tables S2 and S3. Multiplex PCR was conducted in a 25 μl total volume of reaction system containing 1 μl genomic DNA, 2 μl 1 × buffer, 0.6 μl Mg^2+^, 2 μl dNTP, 0.2 μl DNA polymerase, 2 μl Primer mix, and 12.2 μl ddH_2_O. Multiplex PCR was carried out under the following conditions: initial denaturation at 95 °C for 2 min, followed by 40 cycles (94 °C for 90 s, 53 °C for 1.5 min, 65 °C for 30 s), and terminal extension 65 °C for 10 min. After PCR amplification, LDR was performed with a 10 μl volume of reaction system comprising 4 μl PCR product, 1 μl 1 × buffer, 1 μl Probe mix (2 pmol/μl), 0.05 μl Taq DNA ligase, and 4 μl ddH_2_O with 40 production cycles of pre-denaturation at 95 °C for 2 min, annealing at 94 °C for 15 s, and extension at 50 °C for 25 s. The genotypes of the complement *C3* SNPs were identified by an ABI PRISM 3730 sequencer, and the data were analysed by Genemapper software. To ensure the accuracy of the results, approximately 2 % of samples were selected randomly for sequencing.

### Serum lipid and C3 levels

The lipid level detection methods, including TC, triglyceride (TG), high density lipoprotein cholesterol (HDL-C) and LDL-C, apolipoprotein A1 (Apo A1) and Apo B, were described in our previous study [15]. The serum C3 levels were detected by the radio-immunoassay method, according to the instructions for the kit (Roche Diagnosis Co., Ltd., German).

### Statistical analysis

Continuous variables with normal distribution were presented as the means ± standard deviation (SD) and compared with an independent-sample *t*-test or ANOVA test, otherwise presented as median [quartile ranges (QR)] and compared by using Mann–Whitney *U* or Kruskal-Wallis *H* test among groups. Hardy-Weinberg equilibrium was calculated by the Chi-square (χ^2^) test. The differences of allelic and genotypic frequencies between CAD patients and controls were also examined by the Chi-square test. Codominant, dominant and recessive models were used for multiple comparisons. In multiple comparisons, Bonferroni correction was performed to assess the effect of genetic polymorphisms on CAD (Bonferroni corrected P, Pc = P*8) and lipid profiles (Pc = P*16). The association between the genotypes and CAD risk was evaluated by calculating the values of the odds ratios (ORs) and 95% confidence intervals (CIs). Unconditional logistic regression analysis, with adjustment for risk factors of CAD (age, gender, smoking, EH, DM and dyslipidaemia), was also used to analyse the association between the risk for CAD and tagSNPs. Linkage disequilibrium analysis and haplotype analysis were assessed by Haploview 4.2 software package (version 4.2). All statistical analyses were performed with SPSS software (version 17.0, SPSS Inc., Chicago, Illinois, USA). A two-sided *P* value less than 0.05 was considered as statistically significant.

## Results

### Clinical characteristics of subjects

The clinical characteristics of CAD patients and controls are summarized in Table 1. In comparison with controls, patients were older. The prevalence of males and rates of essential hypertension (EH), diabetes mellitus (DM) and smoking were higher among patients than among controls. In lipid profiles, CAD patients had higher TC, triglyceride (TG), LDL-C and ApoB levels than controls. On the contrary, high-density lipoprotein cholesterol (HDL-C) and ApoA1 levels were lower in patients than in controls.

**Table 1:** Clinical characteristics between CAD and control groups

Characteristics	CAD (*n* = 580)	Control (*n* = 437)	*P*
Age (year), mean (SD)	64.16 ± 9.93	61.26 ± 9.35	< 0.001
Gender, male (%)	410 (70.69)	227 (51.95)	< 0.001
EH, n (%)	420 (72.41)	254 (58.12)	< 0.001
DM, n (%)	148 (25.52)	61 (13.96)	< 0.001
Smoking, n (%)	223 (38.44)	109 (24.94)	< 0.001
TC (mmol/l), mean (SD)	4.60 ± 1.06	4.43 ± 0.95	0.012
TG (mmol/l), mean (SD)	1.47(1.06–2.10)	1.36(0.96–2.02)	0.032
HDL-C (mmol/l), mean (SD)	1.08 ± 0.27	1.17 ± 0.31	< 0.001
LDL-C (mmol/l), mean (SD)	2.96 ± 0.95	2.66 ± 0.80	< 0.001
ApoA1 (g/L), mean (SD)	1.18 ± 0.22	1.24 ± 0.24	< 0.001
ApoB (g/L), mean (SD)	0.97 ± 0.29	0.91 ± 0.26	< 0.001

*CAD* Coronary artery disease, *TC* Total cholesterol, *TG* Triglyceride, *HDL-C* High density lipoprotein cholesterol, *LDL-C* Low density lipoprotein cholesterol, *Apo* Apolipoprotein, *EH* Essential hypertension, *DM* Diabetes mellitus, *SD* Standard deviation

### Serum C3 levels and the risk of CAD

A total of 143 samples including 73 controls (43 males and 30 females, mean age of 62.49 ± 8.43 years) and 70 CAD patients (47 males and 23 females, mean age of 65.00 ± 10.24 years) were randomly selected to detect the serum C3 levels. There were no significant differences in the ages and gender between the two groups (*P* > 0.05). The C3 levels were positively associated with the LDL-C levels (r = 0.269, *P* = 0.001). Compared with those in controls, the C3 levels in CAD patients were significantly higher (Control: 0.94 + 0.14 g/l; CAD: 1.10 + 0.19 g/l, t = 5.910, *P* < 0.001). Logistic regression analysis, with adjustment for gender, age, smoking, EH, DM and lipid profiles, showed that the C3 levels remained significantly associated with the risk of CAD (*P* < 0.001).

### Association of *C3* tagSNPs and the risk of CAD

All genotypic frequencies in controls, except rs2230205, were in accordance with Hardy-Weinberg equilibrium. The distributions of the allelic and genotypic frequencies of *C3* polymorphisms in CAD patients and controls are shown in Table 2. No significant differences in the genotype and allele frequencies were observed between patients and controls (Bonferroni corrected P, Pc > 0.05). Codominant, dominant and recessive models were used for multiple comparisons. In multiple comparisons, no significant association of *C3* tagSNPs and CAD risk was found after adjusting for age, gender, EH, DM, smoking and lipid profiles (Additional file 1: Table S4).

**Table 2:** Distribution of the allelic and genotypic frequencies of *C3* polymorphisms in CAD and controls

Tag SNPs	Genotypes/ Allele	CAD	Control	χ^2^	*P*
rs2230205	GG	149	106	1.698	0.428
(*n* = 1013)	GA	293	240		
	AA	134	91		
	G	591	452	0.034	0.858
	A	561	422		
rs2287848	CC	416	331	2.441	0.295
(*n* = 1014)	CT	153	98		
	TT	8	8		
	C	985	760	1.062	0.332
	T	169	114		
rs2250656	AA	347	261	0.335	0.846
(*n* = 926)	AG	162	114		
	GG	23	19		
	A	856	636	0.019	0.906
	G	208	152		
rs7257062	TT	301	244	3.154	0.207
(*n* = 1015)	TC	238	156		
	CC	40	36		
	T	840	644	0.437	0.512
	C	318	228		
rs11672613	TT	185	130	1.097	0.578
(*n* = 993)	TC	283	215		
	CC	97	83		
	T	653	475	1.047	0.315
	C	477	381		
rs2277984	AA	133	118	2.202	0.332
(*n* = 943)	AG	295	209		
	GG	109	79		
	A	561	445	1.226	0.284
	G	513	367		
rs344555	GG	273	190	1.108	0.575
(*n* = 872)	GA	197	145		
	AA	35	32		
	G	743	525	0.890	0.355
	A	267	209		
rs2241393	GG	194	170	4.639	0.098
(*n* = 1015)	GC	287	209		
	CC	98	57		
	G	675	549	4.529	0.035
	C	483	323		

### Association of *C3* tagSNPs and lipid profiles

Table 3 shows the association of *C3* tagSNPs and lipid profiles, after Bonferroni correction of *P*-values, the minor T allele of rs2287848 was associated with low ApoA1 levels in controls (Pc = 0.032).

**Table 3:** Associations of the tag SNPs genotypes and serum lipid levels in the CAD patients and controls

TagSNPs	Groups	Genotypes	TC (mmol/l)	TG(mmol/l)	HDL-C(mmol/l)	LDL-C(mmol/l)	ApoA1(g/l)	ApoB (g/l)
rs2230205	CAD	GG	4.468 ± 1.133	1.330(0.975–2.125)	1.104 ± 0.270	2.822 ± 0.999	1.193 ± 0.222	0.952 ± 0.330
		GA	4.653 ± 1.046	1.490(1.070–2.105)	1.066 ± 0.257	3.014 ± 0.948	1.174 ± 0.225	0.984 ± 0.285
		AA	4.612 ± 1.000	1.490(1.128–2.100)	1.071 ± 0.296	2.991 ± 0.888	1.169 ± 0.215	0.978 ± 0.256
		P	0.218	0.252	0.369	0.121	0.617	0.533
	Control	GG	4.375 ± 0.928	1.415(0.988–2.148)	1.165 ± 0.283	2.593 ± 0.763	1.243 ± 0.226	0.893 ± 0.247
		GA	4.504 ± 0.995	1.345(0.930–1.958)	1.165 ± 0.307	2.718 ± 0.837	1.242 ± 0.248	0.922 ± 0.282
		AA	4.317 ± 0.860	1.340(0.940–1.930)	1.169 ± 0.358	2.566 ± 0.724	1.218 ± 0.244	0.875 ± 0.220
		P	0.218	0.315	0.995	0.196	0.704	0.294
rs2287848	CAD	CC	4.578 ± 1.047	1.470(1.080–2.235)	1.071 ± 0.265	2.939 ± 0.943	1.183 ± 0.225	0.977 ± 0.296
		CT	4.626 ± 1.089	1.450(1.005–1.895)	1.084 ± 0.280	2.987 ± 0.974	1.161 ± 0.216	0.969 ± 0.283
		TT	4.914 ± 1.236	1.135(0.953–1.643)	1.274 ± 0.345	3.233 ± 1.028	1.246 ± 0.184	0.941 ± 0.194
		P	0.617	0.165	0.100	0.617	0.391	0.913
	Control	CC	4.384 ± 0.894	1.340(0.950–1.960)	1.157 ± 0.311	2.631 ± 0.730	1.219 ± 0.234	0.887 ± 0.236
		CT	4.627 ± 1.129	1.430(0.990–2.180)	1.206 ± 0.320	2.736 ± 1.005	1.307 ± 0.255	0.971 ± 0.334
		TT	4.120 ± 0.741	1.465(1.038–1.998)	1.035 ± 0.240	2.684 ± 0.673	1.113 ± 0.207	0.845 ± .0.141
		P	0.054	0.410	0.199	0.522	**0.002**	0.018
rs2250656	CAD	AA	4.653 ± 1.059	1.510(1.100–2.270)	1.079 ± 0.265	3.000 ± 0.951	1.179 ± 0.206	0.983 ± 0.288
		AG	4.544 ± 1.091	1.370(0.978–2.030)	1.072 ± 0.274	2.924 ± 0.962	1.178 ± 0.241	0.973 ± 0.304
		GG	4.396 ± 0.867	1.350(1.030–1.750)	1.157 ± 0.297	2.684 ± 0.790	1.179 ± 0.195	0.867 ± 0.220
		P	0.347	0.315	0.360	0.253	1.000	0.177
	Control	AA	4.400 ± 0.894	1.370(0.990–1.470)	1.157 ± 0.300	2.648 ± 0.750	1.217 ± 0.232	0.898 ± 0.245
		AG	4.557 ± 1.038	1.305(0.893–1.990)	1.191 ± 0.346	2.763 ± 0.916	1.266 ± 0.254	0.915 ± 0.268
		GG	4.196 ± 1.156	1.230(0.870–1.600)	1.197 ± 0.232	2.427 ± 0.676	1.262 ± 0.256	0.801 ± 0.340
		P	0.183	0.062	0.561	0.175	0.177	0.205
rs7257062	CAD	TT	4.612 ± 1.078	1.450(1.070–2.095)	1.085 ± 0.275	2.987 ± 0.990	1.187 ± 0.225	0.977 ± 0.293
		TC	4.615 ± 1.053	1.470(1.050–2.110)	1.065 ± 0.268	2.947 ± 0.915	1.165 ± 0.217	0.979 ± 0.293
		CC	4.378 ± 0.923	1.400(1.045–2.193)	1.086 ± 0.237	2.804 ± 0.846	1.194 ± 0.230	0.927 ± 0.266
		P	0.398	0.742	0.686	0.506	0.484	0.559
	Control	TT	4.483 ± 0.987	1.440(0.993–2.315)	1.152 ± 0.299	2.699 ± 0.849	1.242 ± 0.230	0.930 ± 0.283
		TC	4.404 ± 0.893	1.245(0.923–1.703)	1.186 ± 0.331	2.641 ± 0.696	1.241 ± 0.251	0.8790.282
		CC	4.222 ± 0.987	1.540(0.850–2.208)	1. 168 ± 0.322	2.423 ± 0.844	1.184 ± 0.278	0.860 ± 0.220
		P	0.276	0.015	0.570	0.149	0.388	0.960
rs11672613	CAD	TT	4.629 ± 1.183	1.300(1.020–1.890)	1.117 ± 0.263	3.031 ± 1.104	1.195 ± 0.203	0.966 ± 0.295
		TC	4.611 ± 0.978	1.560(1.130–2.300)	1.055 ± 0.263	2.941 ± 0.837	1.176 ± 0.233	0.990 ± 0.288
		CC	4.500 ± 1.058	1.490(0.995–2.150)	1.067 ± 0.292	2.884 ± 0.949	1.148 ± 0.202	0.940 ± 0.284
		P	0.596	0.004	0.044	0.419	0.234	0.318
	Control	TT	4.460 ± 1.065	1.335(0.988–2.155)	1.158 ± 0.296	2.705 ± 0.983	1.242 ± 0.219	0.921 ± 0.280
		TC	4.441 ± 0.934	1.310(0.960–1.900)	1.174 ± 0.316	2.656 ± 0.695	1.248 ± 0.253	0.907 ± 0.271
		CC	4.350 ± 0.829	1.480(0.930–2.130)	1.144 ± 0.327	2.580 ± 0.746	1.194 ± 0.244	0.874 ± 0.204
		P	0.692	0.412	0.784	0.545	0.217	0.446
rs2277984	CAD	AA	4.542 ± 1.006	1.350(1.015–1.990)	1.059 ± 0.250	2.976 ± 0.905	1.148 ± 0.239	0.942 ± 0.279
		AG	4.599 ± 1.022	1.530(1.080–2.300)	1.073 ± 0.265	2.941 ± 0.916	1.179 ± 0.209	0.988 ± 0.287
		GG	4.694 ± 1.245	1.620(1.070–2.050)	1.109 ± 0.290	2.981 ± 1.076	1.217 ± 0.212	0.985 ± 0.315
		P	0.543	0.032	0.327	0.901	0.049	0.301
	Control	AA	4.424 ± 1.090	1.320(0.905–1.945)	1.174 ± 0.316	2.704 ± 0.982	1.242 ± 0.240	0.907 ± 0.289
		AG	4.420 ± 0.882	1.340(0.975–1.965)	1.178 ± 0.332	2.623 ± 0.694	1.245 ± 0.244	0.890 ± 0.245
		GG	4.533 ± 0.939	1.450(0.940–2.170)	1.150 ± 0.256	2.708 ± 0.758	1.228 ± 0.241	0.929 ± 0.262
		P	0.646	0.538	0.790	0.585	0.875	0.537
rs344555	CAD	GG	4.600 ± 0.990	1.440(1.075–2.070)	1.075 ± 0.276	2.973 ± 0.873	1.168 ± 0.213	0.974 ± 0.287
		GA	4.541 ± 0.988	1.560(1.045–2.300)	1.071 ± 0.260	2.883 ± 0.896	1.179 ± 0.220	0.962 ± 0.273
		AA	5.517 ± 31.687	1.490(1.190–2.390)	1.141 ± 0.274	3.261 ± 1.491	1.267 ± 0.223	1.115 ± 0.438
		P	0.005	0.272	0.355	0.084	0.041	0.017
	Control	GG	4.406 ± 1.029	1.305(0.938–2.013)	1.152 ± 0.296	2.681 ± 0.895	1.224 ± 0.239	0.895 ± 0.275
		GA	4.440 ± 0.828	1.320(0.970–1.945)	1.203 ± 0.355	2.643 ± 0.677	1.252 ± 0.238	0.880 ± 0.213
		AA	4.334 ± 0.898	1.260(0.948–1.930)	1.102 ± 0.367	2.584 ± 0.720	1.171 ± 0.264	0.908 ± 0.252
		P	0.838	0.772	0.158	0.785	0.200	0.790
rs2241393	CAD	GG	4.661 ± 0.989	1.530(1.128–2.233)	1.078 ± 0.260	3.001 ± 0.944	1.200 ± 0.238	0.994 ± 0.303
		GC	4.571 ± 1.126	1.440–2.060	1.076 ± 0.291	2.958 ± 1.010	1.157 ± 0.211	0.966 ± 0.290
		CC	4.535 ± 0.988	1.415(0.960–2.280)	1.077 ± 0.224	2.861 ± 0.765	1.195 ± 0.216	0.959 ± 0.267
		P	0.541	0.227	0.995	0.496	0.076	0.494
	Control	GG	4.511 ± 1.031	1.405(0.970–2.115)	1.157 ± 0.295	2.742 ± 0.895	1.250 ± 0.232	0.928 ± 0.280
		GC	4.362 ± 0.887	1.330(0.985–2.045)	1.171 ± 0.332	2.581 ± 0.720	1.236 ± 0.243	0.889 ± 0.261
		CC	4.464 ± 0.955	1.360(0.850–1.910)	1.168 ± 0.292	2.670 ± 0.754	1.201 ± 0.266	0.903 ± 0.203
		P	0.313	0.511	0.902	0.146	0.421	0.354

*CAD* Coronary artery disease, *TC* Total cholesterol, *TG* Triglyceride, *HDL-C* High density lipoprotein cholesterol, *LDL-C* Low density lipoprotein cholesterol

### Association of and *C3* tagSNPs and serum C3 levels

No significant association between *C3* tagSNPs and C3 levels was found in the whole population (data not shown). Stratified analyses suggested that *C3* polymorphisms were not associated with C3 levels in either cases or controls.

### Linkage disequilibrium and haplotype analysis

**Fig. 1:**
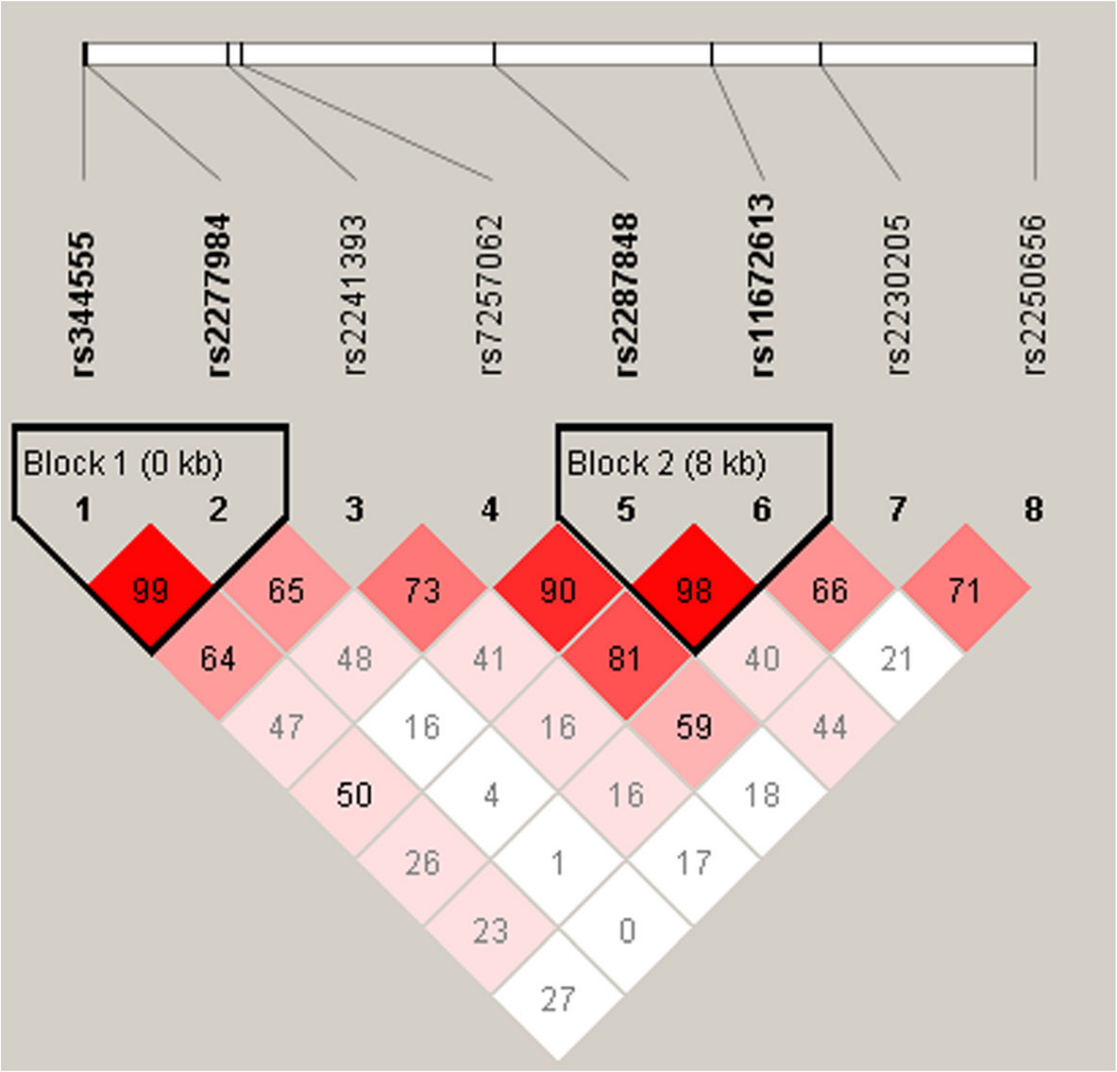
Haplotype block map for tag SNPs of *C3* gene in the whole population

Additional file 1 Table S5 lists the result of the linkage disequilibrium and haplotype analyses. A total of two haplotype blocks (Block1: rs344555-rs2277984, Block 2: rs2287848-rs11672613) and six haplotypes were established (Fig. 1). Haplotype analysis indicated that there were no significant associations between haplotypes and the risk of CAD (all Pc > 0.05).

## Discussion

To our knowledge, this is the first study to evaluate the relationship between tagSNPs in the *C3* gene and the lipid levels and CAD risk in a Chinese population. In the present study, we found that *C3* polymorphisms were associated with lipid levels, but not the risk of CAD.

Complement activation is fundamental to the immune defence of the host. But if the complement component becomes excessive activation, it can lead to various diseases including inflammatory and cardiovascular diseases. C3 has been identified as the central component of the complement system and plays an important role in the pathogenesis of cardiovascular diseases [8]. Cohort studies of the general population demonstrated that high concentrations of complement C3 were associated with diabetes incidence and an increased risk of diabetic microvascular disease [16, 17]. In a case-control study, the serum levels of C3 and C4 were significantly increased in acute myocardial infarction patients and stable angina patients compared with controls [18]. Dyslipidemia, such as high LDL-C and low HDL-C, plays a crucial role in the occurrence and development of CAD and, has been widely accepted. In recent years, numerous studies have demonstrated that C3 is strongly associated with lipid levels and CAD risk [18, 19]. Inhibiting the interaction between C3 and fibrinogen could reduce cardiovascular events in diabetic patients [20]. In our study, we found that the C3 level was positively associated with the LDL-C levels and risk of CAD, which was consistent with previous results.

Recently, studies suggested that variations in the *C3* gene could result in a change in the C3 concentration [21]. In 2009, Phillips detected 11 tagSNPs in *C3* in French people with metabolic syndrome. They found that rs2250656 A allele carriers had obviously higher C3 levels than those with the GG genotype [22]. Another study conducted in European systemic lupus erythematosus families revealed that the rs344555 polymorphism was strongly related to the level of C3 and that the rs2277984 polymorphism was weakly correlated with C3 level [23]. Moreover, the variants in the *C3* gene might influence the activation pathway by changing the molecular structure of C3, in addition to changing the C3 level [24]. Eight SNPs were selected as tagSNPs in our study. Interestingly, no association between *C3* polymorphisms and the C3 level was found in the study, which was inconsistent with previous studies. The reasons for this discrepancy might be partly due to the different ethnicities, environmental factors and our relatively small sample size. In addition, a GWAS on complement activation (defined as serum C3d-to-C3 ratio) had been published in patients with AMD recently [25]. It might be interesting to look if *C3* polymorphisms were related to the C3d-to-C3 ratio and the CAD risk in our next study.

Previous studies have shown that complement *C3* SNPs were significantly associated with the lipid levels and risk of CAD in different populations [11, 22, 26]. In 2009, Phillips et al. revealed that the polymorphism rs2250656 (in the 2nd intron) was associated with metabolic syndrome and lipid levels. Individuals with a C-allele had lower HDL-C levels and higher TG levels than those with a GG genotype [22]. As early as 1972, Dissing et al. found that the *C3* gene polymorphism was associated with atherosclerotic disease in the elderly in a population in Copenhagen [26]. Kristensen et al. [27] found that C3F polymorphisms were associated with the incidence of CAD in patients with high blood pressure and suggested that the C3F allele might speed up the process of artery hardening in hypertension patients. *C3* gene polymorphisms might effect on the express of C3, which interferes with the metabolism of lipids, and leads to the presence of CAD.

Although many studies on the relationship between *C3* polymorphisms and CAD susceptibility have been conducted to date, the results are inconsistent. Some studies suggested that the C3F polymorphism was not associated with CAD risk [28]. In the present study, we explored the relationship between variants of the *C3* gene and the susceptibility to CAD and lipid levels by tagSNPs in a Chinese population. In recent years, two studies were conducted to explore the association between polymorphisms of the Complement 3 gene and diseases in Chinese Han populations [29, 30]. We found the frequencies of alleles in controls in the present study were similar to those in their studies. The results of the present study revealed that *C3* polymorphisms were associated with lipid levels, but the genotype and allele distributions in *C3* tagSNPs were not significantly different between CAD patients and controls.

There were several potential limitations in the present study. First, the tagSNPs were selected using MAF ≥ 0.1 and r^2^ ≥ 0.8 as a cutoff, these thresholds might omit some significant but rare variations. Second, the sample size was relatively small. Therefore, future studies with a larger sample size should be conducted to confirm the findings of the current study. Third, diagnosis of control subjects was mainly according to the results of coronary angiography, potentially resulting in selection bias. Further studies are needed to evaluate the effect of *C3* SNPs and serum C3 levels and their contribution to CAD.

## Conclusions

The study evaluated the relationship between tagSNPs in *C3* gene and lipid levels and CAD risk. The results showed that *C3* gene polymorphisms were associated with lipid levels, but not CAD susceptibility in the Chinese population.

## Supplementary information

**Additional file 1: Table S1.** TagSNPs in *C3* gene summary of all study participants. **Table S2.** Primers of *C3* tagSNPs used in the PCR. **Table S3.** Probes of *C3* tagSNPs used in the LDR. **Table S4.** Associations of *C3* tagSNPs and CAD risk in different comparison models. **Table S5.** Haplotype analyses in CAD patients and controls.

## Data Availability

The datasets used and/or analyzed during the current study are available from the corresponding author on reasonable request.

## Abbreviations

Apo: Apolipoprotein
CAD: Coronary artery disease
DM: Diabetes mellitus
EH: Essential hypertension
HDL-C: High density lipoprotein cholesterol
LDL-C: Low density lipoprotein cholesterol
TC: Total cholesterol
TG: Triglyceride
