# Supplementary material for: Complement C3 gene polymorphisms are associated with lipid levels, but not the risk of coronary artery disease: a case-control study
Source: Lipids Health Dis. 2019 Dec 11;18:217. doi: 10.1186/s12944-019-1163-8 (PMC6905069; doi:10.1186/s12944-019-1163-8)
Supplement: Supplementary file 1 — Additional file 1: Table S1. TagSNPs in C3 gene summary of all study participants. Table S2. Primers of C3 tagSNPs used in the PCR. Table S3. Probes of C3 tagSNPs used in the LDR. Table S4. Associations of C3 tagSNPs and CAD risk in different comparison models. Table S5. Haplotype analyses in CAD patients and controls. [file 12944_2019_1163_MOESM1_ESM.doc]

**Supplementary Table 1. TagSNPs in *C3* gene summary of all study participants**

| SNPs | Chr | Position | Functional Consequence | Allele | Number | MAF  (CAD) | MAF  (controls) | OR (95%CI) | *P* | *P **  (HWE) |
| --- | --- | --- | --- | --- | --- | --- | --- | --- | --- | --- |
| rs2250656 | 19 | 6718523 | intron variant | A/G | 926 | 19.55% | 19.29% | 1.017(0.805-1.283) | 0.906 | 0.037 |
| rs344555 | 19 | 6679349 | intron variant | G/A | 872 | 26.44% | 28.47% | 0.903(0.730-1.117) | 0.355 | 0.812 |
| rs11672613 | 19 | 6705235 | intron variant | T/C | 993 | 42.21% | 44.51% | 0.911(0.761-1.089) | 0.315 | 0.160 |
| rs2287848 | 19 | 6696331 | intron variant | C/T | 1014 | 14.64% | 13.04% | 1.144(0.886-1.477) | 0.332 | 0.125 |
| rs7257062 | 19 | 6685934 | intron variant | T/C | 1015 | 27.46% | 26.15% | 1.069(0.877-1.304) | 0.512 | 0.726 |
| rs2230205 | 19 | 6709693 | synonymous codon | G/A | 1013 | 48.70% | 48.28% | 1.017(0.853-1.212) | 0.858 | 0.430 |
| rs2277984 | 19 | 6679500 | intron variant | A/G | 943 | 47.77% | 45.20% | 1.109(0.923-1.331) | 0.284 | 0.565 |
| rs2241393 | 19 | 6685293 | intron variant | G/C | 1015 | 41.07% | 37.04% | 1.216(1.015-1.457) | 0.035 | 0.562 |

SNP, single-nucleotide polymorphism; CAD, coronary artery disease; Chr, Chromosome; HWE, Hardy-Weinberg Equilibrium; *, HWE in controls; OR, odds ratio; CI, confidence interval; MAF, minor allele frequency.

**Supplementary Table 2. Primers of *C3* tagSNPs used in the PCR**

LDR, ligase detection reaction

| Primer name | F sequence(5’-3’) | R sequence(5’-3’) | PCR length |
| --- | --- | --- | --- |
| rs2250656 | CCTCGCACCTCCTTCACATG | GTCAGGGGTCTGTTCCAAGG | 140 |
| rs344555 | ACAAGCTCTGCCGTGATGAA | CCCTTGGTATCCCCTGGGTA | 148 |
| rs11672613 | TACAGCCCACCATCCATGTG | TCTCCTTCACAAAGAGGCGA | 103 |
| rs2287848 | ACACCGAGTCTGAGACCAGA | AAGGGGAAGAGAAAGGTGCG | 138 |
| rs7257062 | TGATCTAGACACCTGGGGCA | CTTCATTCCCACGAGCCAGT | 144 |
| rs2230205 | GCAGCAGATGACCCTGAAGA | GCCTCTTCTCAGCAGCCTTG | 148 |
| rs2277984 | CATGGTCTTTGGAGGGAGGC | ATCACGGCAGAGCTTGTTCA | 125 |
| rs2241393 | GCATCTCCAAGTCCTGGTGT | TGGGACATCAAAATGTGGCCT | 127 |

**Supplementary Table 3. Probes of *C3* tagSNPs used in the LDR**

| Probe name | Sequence (5'-3') | LDR length |
| --- | --- | --- |
| rs2230205_modify | P-GTCAGTTTGTTCTTCTTATTTTTTTTTTTTTTTTTTTT-FAM |  |
| rs2230205_A | TTTTTTTTTTTTTTTTCACTGGCCCTTACCTTACTCTGT | 77 |
| rs2230205_G | TTTTTTTTTTTTTTTTTTCACTGGCCCTTACCTTACTCTGC | 79 |
| rs2287848_modify | P-TCCATGCCCTCCTGGGACCCTTTTTTTTTTTTTTTTTTTT-FAM |  |
| rs2287848_C | TTTTTTTTTTTTTTTTTTGAATGAGATGGAATTTGGCTCCG | 81 |
| rs2287848_T | TTTTTTTTTTTTTTTTTTTTGAATGAGATGGAATTTGGCTCCA | 83 |
| rs2250656_modify | P-CCGAGGTGGCCGTTTTGGGGTTTTTTTTTTTTTTTTTTTTTTTT-FAM |  |
| rs2250656_A | TTTTTTTTTTTTTTTTTTTTTTGCCTGCCCATTATTCTTGGTCTT | 89 |
| rs2250656_G | TTTTTTTTTTTTTTTTTTTTTTTTGCCTGCCCATTATTCTTGGTCTC | 91 |
| rs7257062_modify | P-TACTCCAAGCTGTGTGTTGATTTTTTTTTTTTTTTTTTTTTTTTTT-FAM |  |
| rs7257062_C | TTTTTTTTTTTTTTTTTTTTTTTTTAGGTTCTAGAATACAATCTGGG | 93 |
| rs7257062_T | TTTTTTTTTTTTTTTTTTTTTTTTTTTAGGTTCTAGAATACAATCTGGA | 95 |
| rs11672613_modify | P-AAGTCTGGTCTACTGAAATATTTTTTTTTTTTTTTTTTTTTTTTTTTTTT-FAM |  |
| rs11672613_C | TTTTTTTTTTTTTTTTTTTTTTTTTTTTGGTCACTGGGAAAATTAGACAAG | 101 |
| rs11672613_T | TTTTTTTTTTTTTTTTTTTTTTTTTTTTTTGGTCACTGGGAAAATTAGACAAA | 103 |
| rs2277984_modify | P-GGGCAGATGTGATGTGAAGATTTTTTTTTTTTTTTTTTTTTTTTTTTTTTTT-FAM |  |
| rs2277984_A | TTTTTTTTTTTTTTTTTTTTTTTTTTTTTTAACCGGGTACAGCTTTCCTCTGT | 105 |
| rs2277984_G | TTTTTTTTTTTTTTTTTTTTTTTTTTTTTTTTAACCGGGTACAGCTTTCCTCTGC | 107 |
| rs344555_modify | P-GACCTGGGTAAGTGTGGCTTTTTTTTTTTTTTTTTTTTTTTTTTTTTTTTTTTT-FAM |  |
| rs344555_A | TTTTTTTTTTTTTTTTTTTTTTTTTTTTTTTTGGGTCCCTGACCATGGGATAGAT | 109 |
| rs344555_G | TTTTTTTTTTTTTTTTTTTTTTTTTTTTTTTTTTGGGTCCCTGACCATGGGATAGAC | 111 |
| rs2241393_modify | P-TCACTGGACTCTGAGGACATTTTTTTTTTTTTTTTTTTTTTTTTTTTTTTTTTTTT-FAM |  |
| rs2241393_C | TTTTTTTTTTTTTTTTTTTTTTTTTTTTTTTTTTAGTGCAGGGGTGATAACTGGAGG | 113 |
| rs2241393_G | TTTTTTTTTTTTTTTTTTTTTTTTTTTTTTTTTTTTAGTGCAGGGGTGATAACTGGAGC | 115 |

LDR, ligase detection reaction

**Supplementary Table 4. Associations of *C3* tagSNPs and CAD risk in different comparison models**

|  |  | **Total** | **Control** | **CAD** | ***P*** | **OR(95%CI)** | ***P*^a^** | **OR^a^ (95%CI)** |
| --- | --- | --- | --- | --- | --- | --- | --- | --- |
| **rs2230205** | GG | 255 | 106 | 149 |  | 1 |  | 1 |
| (n=1013) | GA | 533 | 240 | 293 | 0.360 | 0.869(0.642-1.175) | 0.117 | 0.767(0.550-1.068) |
|  | AA | 225 | 91 | 134 | 0.803 | 1.048(0.728-1.508) | 0.591 | 0.896(0.600-1.338) |
|  | GA+AA | 758 | 331 | 427 | 0.558 | 0.918(0.688-1.223) | 0.172 | 1.246(0.909-1.709) |
|  | GG+GA | 788 | 346 | 442 | 0.355 | 1.074(0.924-1.248) | 0.682 | 1.072(0.769-1.493) |
| **rs2287848** | CC | 747 | 331 | 416 |  | 1 |  | 1 |
| (n=1014) | CT | 251 | 98 | 153 | 0.145 | 1.242(0.928-1.663) | 0.257 | 1.203(0.874-1.656) |
|  | TT | 16 | 8 | 8 | 0.651 | 0.796(0.295-2.143) | 0.943 | 0.461(0.328-1.656) |
|  | CT+TT | 267 | 106 | 161 | 0.192 | 1.209(0.909-1.606) | 0.283 | 1.186(0.868-1.620) |
|  | CC+CT | 998 | 429 | 569 | 0.575 | 0.868(0.530-1.423) | 0.877 | 0.919(0.314-2.689) |
| **rs2250656** | AA | 608 | 261 | 347 |  | 1 |  | 1 |
| (n=926) | AG | 276 | 114 | 162 | 0.651 | 1.069(0.801-1.426) | 0.459 | 1.127(0.822-1.545) |
|  | GG | 42 | 19 | 23 | 0.770 | 0.911(0.486-1.707) | 0.922 | 1.036(0.516-2.079) |
|  | AG+GG | 318 | 133 | 185 | 0.747 | 1.046(0.795-1.377) | 0.481 | 1.114(0.825-1.506) |
|  | AA+AG | 884 | 375 | 509 | 0.718 | 0.944(0.692-1.289) | 0.994 | 0.997(0.500-1.988) |
| **rs7257062** | TT | 545 | 244 | 301 |  | 1 |  | 1 |
| (n=1015) | TC | 394 | 156 | 238 | 0.114 | 1.237(0.951-1.609) | 0.121 | 1.259(0.941-1.683) |
|  | CC | 76 | 36 | 40 | 0.670 | 0.901(0.557-1.457) | 0.590 | 0.866(0.513-1.461) |
|  | TC+CC | 470 | 192 | 278 | 0.209 | 1.174(0.914-1.507)  90 | 0.235 | 1.181(0.897-1.555) |
|  | TT+TC | 939 | 400 | 539 | 0.420 | 0.908(0.718-1.148) | 0.358 | 0.788(0.473-1.311) |
| **rs11672613** | TT | 315 | 130 | 185 |  | 1 |  | 1 |
| (n=993) | TC | 498 | 215 | 283 | 0.593 | 0.925(0.695-1.231) | 0.875 | 0.975(0.712-1.336) |
|  | CC | 180 | 83 | 97 | 0.296 | 0.821(0.568-1.188) | 0.216 | 0.773(0.514-1.163) |
|  | TC+CC | 678 | 298 | 380 | 0.427 | 0.896(0.684-1.175) | 0.570 | 0.917(0.681-1.236) |
|  | TT+TC | 813 | 345 | 468 | 0.368 | 0.928(0.789-1.092) | 0.187 | 0.785(0.548-1.125) |
| **rs2277984** | AA | 251 | 118 | 133 |  | 1 |  | 1 |
| (n=943) | AG | 504 | 209 | 295 | 0.148 | 1.252(0.923-1.698) | 0.153 | 1.278(0.913-1.788) |
|  | GG | 188 | 79 | 109 | 0.298 | 1.244(0.836-1.792) | 0.369 | 1.212(0.797-1.845) |
|  | AG+GG | 692 | 288 | 404 | 0.140 | 1.245(0.931-1.664) | 0.158 | 1.260(0.914-1.735) |
|  | AA+AG | 755 | 327 | 428 | 0.749 | 1.027(0.873-1.207) | 0.872 | 1.030(0.722-1.469) |
| **rs344555** | GG | 463 | 190 | 273 |  | 1 |  | 1 |
| (n=872) | GA | 342 | 145 | 197 | 0.699 | 0.946(0.712-1.255) | 0.883 | 0.977(0.714-1.336) |
|  | AA | 67 | 32 | 35 | 0.298 | 0.761(0.455-1.273) | 0.103 | 0.626(0.357-1.099) |
|  | GA+AA | 409 | 177 | 232 | 0.504 | 0.912(0.697-1.194) | 0.514 | 0.906(0.674-1.219) |
|  | GG+GA | 805 | 335 | 470 | 0.329 | 0.883(0.688-1.133) | 0.100 | 0.632(0.366-1.093) |
| **rs2241393** | GG | 364 | 170 | 194 |  | 1 |  | 1 |
| (n=1015) | GC | 496 | 209 | 287 | 0.183 | 1.203(0.916-1.580) | 0.184 | 1.225(0.908-1.651) |
|  | CC | 155 | 57 | 98 | 0.037 | 1.507(1.024-2.216) | 0.053 | 1.521(0.995-2.325) |
|  | GC+CC | 651 | 266 | 385 | 0.072 | 1.268(0.979-1.642) | 0.081 | 1.288(0.969-1.710) |
|  | GG+GC | 860 | 379 | 481 | 0.092 | 1.164(0.976-1.389) | 0.126 | 1.354(0.918-1.995) |

CAD, coronary artery disease; OR, odds ratio; CI, confidence interval; ^a^, adjustment with sex, age, DM, EH, smoking and lipid profiles.

**Supplementary Table 5. Haplotype analyses in CAD patients and controls**

| **Blocks** | **Haplotypes** | **Freq** | **CAD** | **Controls** | ***χ*^2^** | ***P*** |
| --- | --- | --- | --- | --- | --- | --- |
| Block 1  (D=0.99, r^2^=0.419) |  |  |  |  |  |  |
| rs344555-rs2277984 | GA | 0.530 | 566.6/525.4 | 446.9.373.1 | 1.275 | 0.2589 |
|  | AG | 0.273 | 288.0/804.0 | 233.8/586.2 | 1.081 | 0.2985 |
|  | GG | 0.196 | 234.9/857.1 | 139.0/681.0 | 6.206 | 0.0127 |
| Block 2  (D=0.98, r^2^=0.118) |  |  |  |  |  |  |
| rs2287848-rs11672613 | CC | 0.432 | 485.5/666.5 | 388.0/484.0 | 1.118 | 0.2902 |
|  | CT | 0.430 | 499.5/652.5 | 371.0/501.0 | 0.134 | 0.7143 |
|  | TT | 0.138 | 167.0/985.0 | 113.0/759.0 | 0.985 | 0.3211 |

CAD, coronary artery disease
